# Challenges and implementation of the German maternity protection act for female medical students in macroscopic anatomical education

**DOI:** 10.3205/zma001310

**Published:** 2020-03-16

**Authors:** Christoph Kulisch, Jana Langheinrich, Evelyn Heuckendorf, Imre Vida, Irene Brunk

**Affiliations:** 1 Charité - Universitätsmedizin Berlin, Institut für Integrative Neuroanatomie, Berlin, Germany; 2 Charité - Universitätsmedizin Berlin, Institut für Funktionelle Anatomie, Berlin, Germany

**Keywords:** maternity protection act, pregnancy, breastfeeding period, women, medical education, medicine, anatomy, anatomical dissection, university education, plastination, body donation, formaldehyde

## Abstract

**Background:** Recent decades have seen controversial discussions on the validity of dissection courses in medical education, with alternative programs tested for various reasons. On April 1, 2015 the classification of formaldehyde as a hazardous substance was upgraded by the EU, leding to some universities precluding the participation of pregnant and breastfeeding students in dissection course. However, the revision to the Maternity Protection Act, implemented in Germany on January 1, 2018, now protects student mothers from being disadvantaged in their studies as a consequence of their pregnancy or breastfeeding. Therefore, universities must offer alternatives to dissection courses using formaldehyde to these female students.

**Project description: **As an alternative to regular dissection courses, which use the abovementioned chemical, the Centre for Anatomy at Charité has opted for developing dedicated courses for student mothers. These new courses use plastinated prosection material instead of formalin-treated cadavers of body donors. As the core of the anatomical education takes place during the third and fourth semester in the current curriculum of human medicine at Charité the alternative courses are limited to those two semesters. Additionally, alternative exams at the end of both semesters had to be developed. The alternative courses were designed to offer pregnant and breastfeeding students a study program as close as possible to the one in which their peers learn human anatomy.

**Results: **For the new courses, plastinates had to be produced and further specimens are still needed. Additionally required sets of bones, models and radiological images were readily available at the Centre for Anatomy. The planning and conceptualization of the courses took half a year of intense preparation. The courses for the third and fourth semester were first running during summer semester 2017. There is a clear demand for courses among pregnant and breastfeeding students. At least 5 student participants per course were registered, corresponding to every fortieth female student in their semester cohorts. The highest number of student participants was 13 in one course so far. The performances of the participants in the anatomical examinations were matching that of students attending the regular courses.

**Discussion: **The alternative macroscopic anatomy courses enable the implementation of the revised Maternity Protection Act. The targeted student group is highly satisfied with the offered alternative courses. Considering the number of participants and their examination performance so far, the Centre for Anatomy regards the efforts involved in planning and implementing the courses as justified. The courses allow pregnant and breastfeeding students to address the same anatomical themes at the same time as their fellow students. However, due to restricted flexibility of plastinates and because students cannot prepare specific anatomical structures independently the scope of topographic learning is limited. That being said, well-produced plastinates can display anatomical structures which often cannot be dissected in regular courses. The alternative macroscopic anatomy courses using plastinates constitute suitable alternatives to the regular dissection courses with formalin-treated cadavers for pregnant and breastfeeding students.

## Zusammenfassung

**Hintergrund: **Während der letzten Jahrzehnte wurde viel und kontrovers über die Notwendigkeit der Dissektionskurse in der Ausbildung angehender Humanmediziner*innen diskutiert. Aus unterschiedlichen Gründen wurden verschiedene Alternativen getestet. Die zum 01.04.2015 geänderte Gefahrenstoffeinstufung von Formaldehyd bewirkte an Universitäten teilweise den Ausschluss von schwangeren und stillenden Studentinnen aus den Präpariersälen. Die in Deutschland zum 01.01.2018 in Kraft getretene Neuregelung des Mutterschutzgesetzes schützt seitdem auch die studentischen Mütter vor nachteiliger Behandlung aufgrund von Schwangerschaft oder Stillen. Deswegen sollten die Universitäten den Studentinnen Ersatzmöglichkeiten anbieten.

**Projektbeschreibung: **Für schwangere und stillende Studentinnen hat sich der Fächerverbund Anatomie als Alternative zu den regulären Präparierkursen für Ersatzkurse mit Plastinaten anstelle von formaldehydfixierten Körpern von Körperspender*innen entschieden und entsprechende Konzepte erarbeitet. Im Modellstudiengang Medizin der Charité findet der Hauptteil der anatomischen Ausbildung im 3. und 4. Fachsemester (FS) statt, weswegen die alternativen Kurse auf diese FS beschränkt sind. Ebenfalls mussten alternative Prüfungen nach beiden FS ermöglicht werden. Die Alternativkurse sollen den Studentinnen möglichst die gleichen Voraussetzungen für das Erlernen der menschlichen Anatomie bieten, wie sie ihre Kommiliton*innen haben.

**Ergebnisse: **Viele Plastinate mussten und einige müssen zurzeit noch neu hergestellt werden. Über einen zusätzlich benötigten Satz an Knochen, Modellen und radiologischen Abbildungen verfügte der Fächerverbund Anatomie bereits. Planung und Kurskonzeptionen bedurften ein halbes Jahr intensiver Ausarbeitungen. Zum Sommersemester 2017 konnten die Kurse des 3. und 4. FS erstmalig durchgeführt werden. Die Nachfrage durch schwangere und stillende Studentinnen ist vorhanden. Mindestens 5 Teilnehmerinnen waren jeweils eingeschrieben, was jeder 40. Studentin im jeweiligen FS entspricht. Das bisherige Maximum eines Kurses lag bei 13 Teilnehmerinnen. Die erbrachten anatomischen Prüfungsleistungen sind annähernd dieselben, wie die der Teilnehmerinnen der regulären Kurse.

**Diskussion: **Das Alternativangebot setzt die Neuregelung des Mutterschutzgesetzes sehr gut um. Die Zufriedenheit bei den entsprechenden Studentinnen ist hoch. Der planerische als auch der praktische Mehraufwand bei der Durchführung der Kurse wird aufgrund der Anzahl der Teilnehmerinnen und der bisherigen Prüfungsleistungen vom Fächerverbund als gerechtfertigt eingestuft. Die Studentinnen haben die Möglichkeit dieselben Themen zeitgleich mit ihren Kommiliton*innen zu behandeln. Das nicht selbstständige, gezielte Präparieren von Körperstrukturen sowie die Inflexibilität der Plastinate stellen Limitationen bezüglich des topografischen Lernens dar. Andererseits können durch gut gearbeitete Plastinate auch Strukturen dargestellt werden, die in den regulären Kursen häufig nicht präpariert werden. Die Ersatzkurse mit Plastinaten stellen für schwangere und stillende Studentinnen eine sehr gute Alternative zu den Dissektionskursen mit formaldehydfixierten Körpern dar.

## 1. Introduction

In Europe, human cadavers have been used for about seven centuries (since the renaissance in Italy) for the anatomical education of medical students [1], [2], [3]. In recent decades, the use of human preparations and cadavers in medical education has come under discussion [2], [4], [5], [6], [7], [8], [9]. Medical training at Charité includes dissection courses with cadavers from body donors. These courses, in combination with other didactic methods implemented in the curriculum, constitute what is considered to be the best teaching strategy [10].

Thorough knowledge of human anatomy is a fundamental requirement for medical practice, not only in surgery, radiology or physical examination, but also in explaining procedures to patients. Some authors claim that anatomical dissection courses are the most widespread and universal characteristic components of medical education [3].

In recent decades, however, the share of practical anatomical teaching performed through dissection courses at medical faculties has decreased due to reorganizations of medical curricula in many countries [4], [5], [11], [12]. In the model degree program of medicine at Charité, dissection courses are taught in the third and fourth semester. These courses are comprised of a number of teaching units (TU, 45 min each) to be studied per week: four a week in the third semester, and two a week in the fourth (for the structure of topics see table 1 (Tab. 1)). This corresponds only to exactly two thirds of the total TUs of anatomical education in the previous classical curriculum. Some prosection courses are included in later semesters.

For over 20 years, the apparent decline in the anatomical knowledge of medical students has been the focal point of international discussions in surgical specialties [13], [14]. Arguments are made that the level of anatomical knowledge necessary for becoming a medical doctor cannot be achieved anymore [15], [16]. A study from 1999 showed that only 29% of new medical residents in the United States had an adequate level of anatomical training [17]. Another study stated that students struggle to learn about physical examinations and diagnoses due to insufficient anatomical knowledge [16].

Two recent legal changes with significant impact on medical education added further aspects to the historical discussion.

The first of these changes pertains to the GHS classification (Globally Harmonized System of Classification and Labeling of Chemicals) of formaldehyde (FA). On April 1, 2015, FA was classified as more hazardous and uprated to category 1B due to its carcinogenicity. Furthermore, as to its Mutagenicity, FA was classified into category 2, as it may also cause genetic defects [18]. As a result, an executive decision was made at Charité to prevent pregnant and breastfeeding students from entering dissection halls to protect their unborn and/or breastfed children. This was decided following occupational, medical and juridical considerations, as well as regarding considerations related to technical aspects of occupational safety. Until 2017 this meant female students had to make up for the dissection courses post-weaning. Alternative ways, other than the regular dissection courses, by which these students could acquire their anatomical knowledge, were needed [19]. However, due to the complexity of themes, the high clinical relevance and the large contingent of TUs, there were no reasonable options available to the students to reduce the delay in their studies. As a result, pregnant and breastfeeding students were delayed in their anatomical studies for up to four semesters or even more, preventing them to complete their third and/or fourth semester, as well as restricting their admission to higher semesters. This unacceptable circumstance, and the additional pressure for these students to catch up on weekly dissection courses and corresponding examinations during subsequent two semesters presented a major disadvantage for pregnant and breastfeeding students.

The second change was the revision to the Maternity Protection Act (Gesetz zur Neuregelung des Mutterschutzrechts) [20] that came into effect on January 1, 2018 in Germany. The Maternity Protection Act, which protects working mothers, was revised to include mothers in higher education and in vocational training. This act ensures that pregnant and breastfeeding women are not disadvantaged because of their circumstances. Higher education institutions and faculties must provide alternatives to courses/programs which mothers cannot attend, unless this is proven not feasible “due to verifiable, disproportionate effort to the institution” [20].

On the basis of these new regulations the Centre for Anatomy at Charité decided to develop and implement alternative macroscopic anatomical courses and examinations for pregnant and breastfeeding students in the third and fourth semester using FA-free plastinates that are acceptable to the female students and reasonable for the Centre for Anatomy.

The revision of the Maternity Protection Act not only affects anatomical education at Charité. Pregnant and breastfeeding students are prevented from attending a number of courses in other fields, such as a practical training for the preparation of blood samples, some bedside teaching units, and part of the courses on pathology. All of these, however, are courses with a much lower amount of TUs, often single classes. The affected students may use their contingent of permissible absences. Establishing alternatives to these courses is therefore viewed as not purposeful and would constitute a disproportional additional effort. Therefore, until September 2019, there were no alternative courses in any other subject fields but anatomy at Charité.

This article outlines the design of alternative macroscopic anatomical courses and presents a viable way of implementing the Maternity Protection Act. The implementation of these courses is discussed in the context of the abovementioned debates.

## 2. Project description

The following pilot project for implementing the Maternity Protection Act was initially approved for 2 years by the Vice Dean for Learning and Teaching at Charité and was running from summer semester 2017 until winter semester 2018/2019. In this pilot project alternatives to formalin-fixed specimens were assessed and then course programs were designed and implemented subsequently. The aim was to develop alternative courses which offer pregnant and breastfeeding students a study program which enables them to acquire anatomical knowledge as efficiently as their fellow students without the opportunity of carrying out anatomical dissections themselves.

### 2.1. Determining possible teaching methods without formalin-fixed wet specimens

The methods for preserving a body are limited. One method is to use a combination of phenol and ethanol [21], [22], which is prohibited in Germany due to health and safety concerns. Conservation with ethanol alone would require extensive refurbishment as well as the purchase of expensive devices. Additionally, cadavers preserved this way are shorter lived than FA-fixed bodies [22]. Furthermore, ethanol based conservation does not ensure the disinfection of bodily fluids [23]. Currently the German Anatomical Society takes the view that an adequate conservation without FA is not viable [24]. Even if there was a safe alternative to FA-fixed cadavers, it is unacceptable for women in advanced stages of their pregnancy to be standing for 90 minutes and work bent over a table for such prolonged periods.

Today, anatomical three-dimensional (3D) prints and computer programs are readily available from commercial providers. Virtual dissection tables are being used in anatomical education, as well as for specific surgical questions [25], [26], and instructional videos have been utilized for decades to transfer anatomical knowledge [6], [27]. However, we considered relying on virtual media alone is not sufficient, as many of the essential aspects of dissection courses include the 3D visual experience, the positional change of different structures to one another and the haptic perception of body structures. Even though the models used in regular courses are 3D and can be handled, they are rather simplified and are not suitable as the only visualization tool. Additionally, the range of models is limited and does not include all body structures.

A further argument against using only these methods and/or materials, alone or in combination, as alternative to regular dissection is that medical faculties would also have to offer alternative examinations to these students. The exams have to be as similar as possible to the standard exams in their implementation and demand the same level of recognition from the students. However, the level of difficulty of a given question is considerably lower if a question is asked on a model rather than on human prosection material (unpublished results). There are similar findings for 3D prints [28] and the same can be expected for schematic illustrations. Students learning with three-dimensional models perform better in examinations than students learning with two-dimensional illustrations [29], [30]. After the third semester in our curriculum a “3D-MC” exam is held. This exam consists of 20 stations with one multiple-choice question referring to a prosection material, model, microscope specimen, or an illustration at each station with one minute to answer each question. The students needed 60% correct answers to pass (i.e. 12 out of 20 points). After the fourth semester an oral exam (10 minutes) takes place with introductory questions from a list of questions referring to prosection materials, models and microscopes. This exam is part of a general exam which consists of 12 sections each lasting 10 minutes. Students can pass the general examination with an average result of 60% (grade 4) or higher. Students can sit these exams either directly after the forth semester or at a later time.

#### 2.2. Plastinates as an option in anatomical education

Another alternative to regular dissection courses is the use of plastinates for the alternative courses and exams. After the plastination process the tissue is suffused with silicone, free of FA and solid. The finished plastinates are safe [31], almost odorless, real and offer a tactile experience. The silicone used in the plastinates (S10, BIODUR, Heidelberg) is a mixture of safe siloxanes, which are polymerized with are crosslinker (S6) and a hardening agent (S3). When handling S3 and S6 personal protective gear should be worn [32] (see also the current MSDS from 2017 and 2018). However, after the plastinates have cured and fully hardened they are safe and can be touched with bare hands [31].

Topographical understanding is better supported by anatomical specimens compared to 3D models, which are in turn better than 2D illustrations [33], [34]. Some universities use only plastinates for anatomical education [15], [35], [36], [37]. A study in the USA showed that dentistry students perform better in exams when they learn with plastinates combined with other new teaching methods in comparison to those attending the cadaver-based dissection course [26].

At the Centre for Anatomy at Charité a basic set of about 50 plastinates (e.g. dissections and cross sections of the extremities, preparations of inner organs, such as heart, lung, stomach, sections of bowel, kidney, liver, bladder, as well as preparations of the head, brain, and neck, cross sections of brains, neck and thorax and preparations of the ligaments of the shoulder, the elbow, the hand, the pelvis, the knee and the foot) was already available. As it was possible to produce further plastinated prosection material, offering and implementing the alternative macroscopic anatomy courses and exams utilizing primarily these FA-free plastinates was considered as the best solution.

All organs for the production of the plastinates, as well as the cadavers for the dissection courses, are from body donors [38], who have provided their personal consent in a written agreement to the use of their body after their death with the Centre for Anatomy. All procedures of handling the corpses are conducted in accordance with the “Law for the regulation of the anatomical dissection” (Sektionsgesetz, SRegG BE) [39] and the “Law on body and burial services” (Bestattungsgesetz, BestattG BE) of the federal state of Berlin, Germany [40]. The plastinates are produced according to the S10 standard silicone technique [31], [41].

#### 2.3. Course design

**Figure 1:**
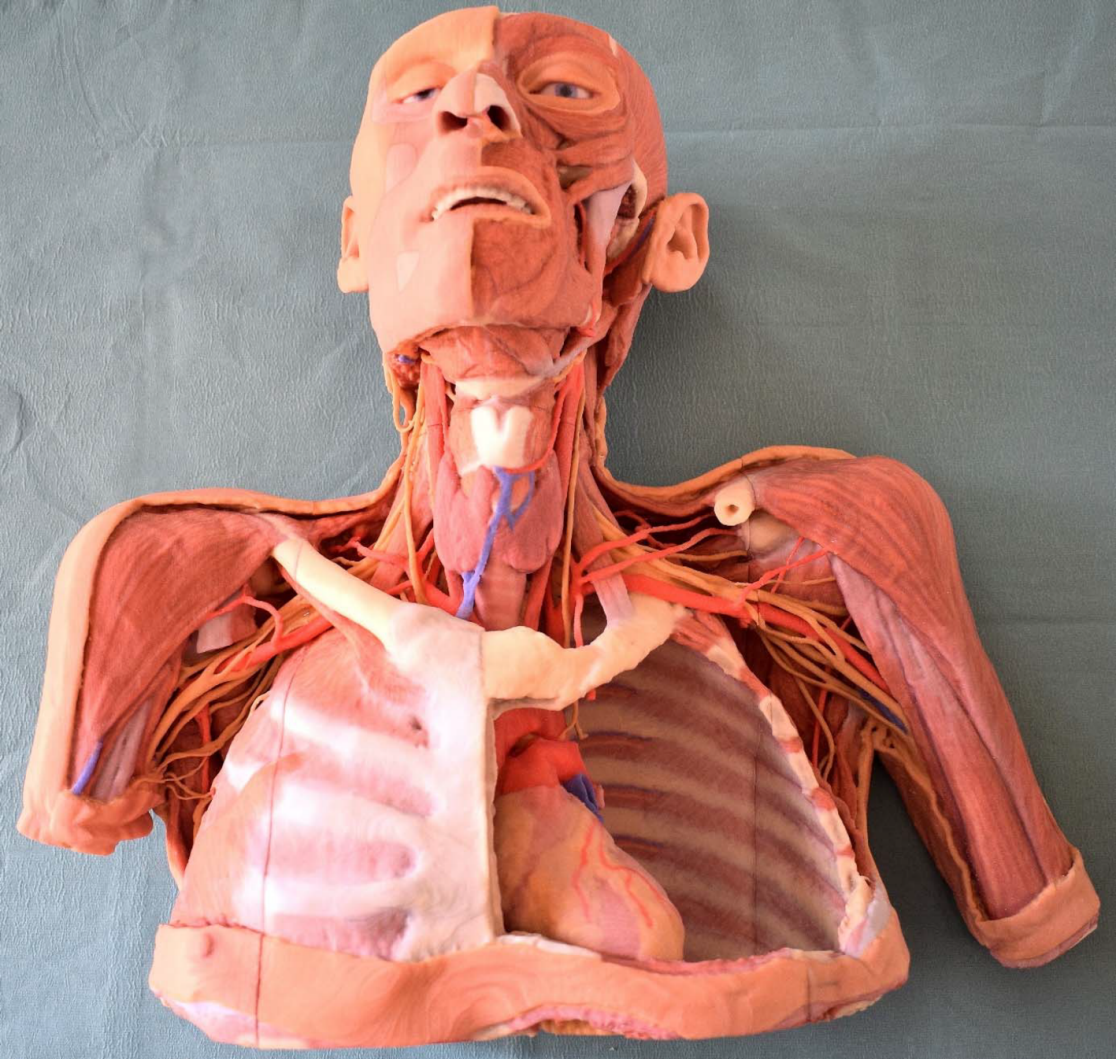
3D print of a dissected thorax with neck, head and the proximal upper extremities made by the company “Erler-Zimmer GmbH & Co.KG”. Large scale (1:1) 3D printed models, like the one shown above, are scanned copies of dissected cadavers which were digitalized and then processed for printing. Such 3D prints can depict the topography very well. While they are light, a major limitation, is that they cannot be modified. In addition to this 3D print (MP 1250), a model of the upper orbit (MP 1675), the female hip (MP 1785), and the lower extremities (MP 1810) were purchased.

**Figure 2:**
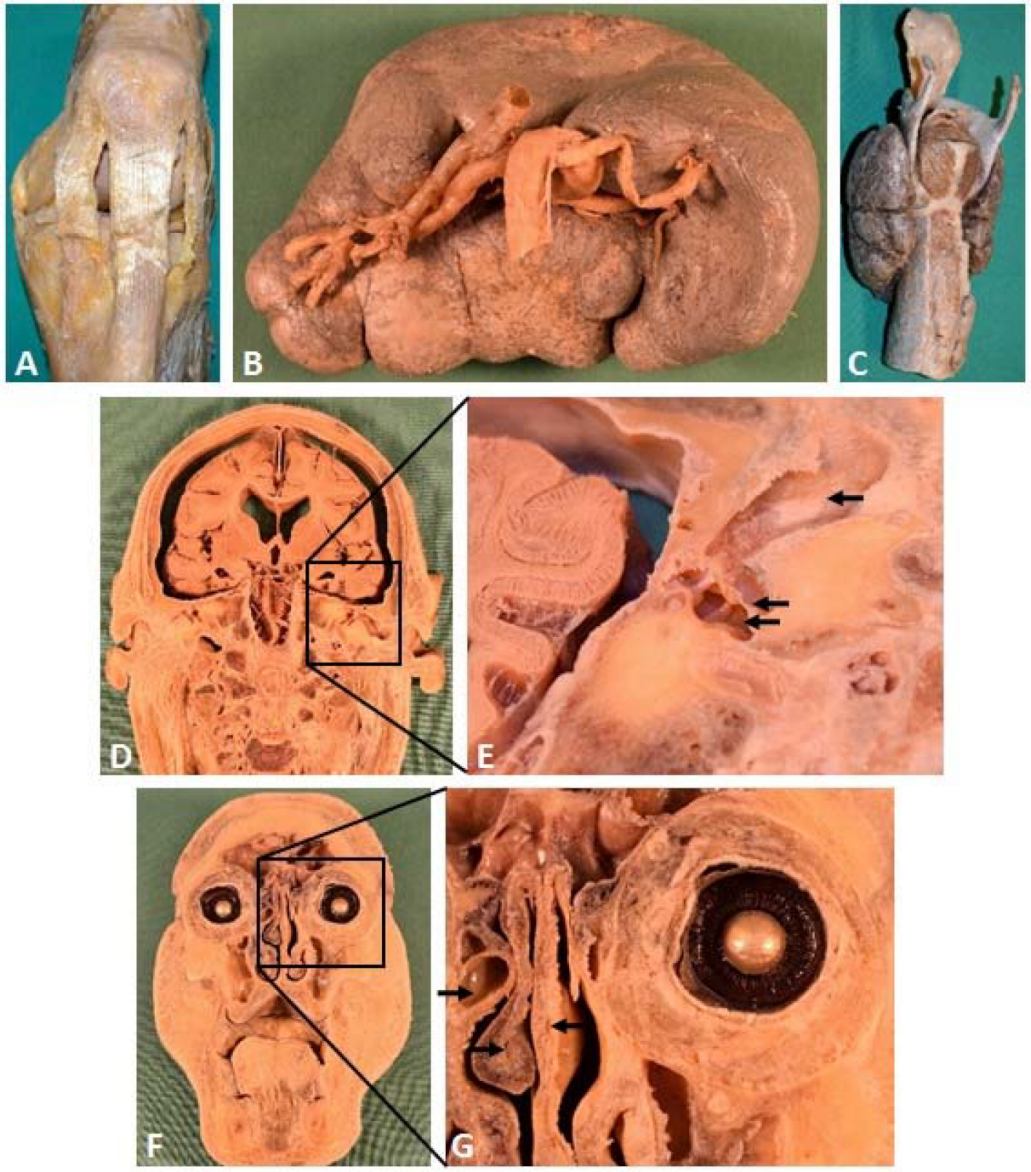
Examples of plastinated specimens which were produced at the Centre for Anatomy for the alternative courses for pregnant and breastfeeding students. A – C show isolated anatomical structures or organs. A) a left knee with tendons and ligaments (Lig. patellae and Retinacula patellae mediale et laterale, prominent); B) an enlarged spleen (splenomegaly) with its hilum shown; C) a larynx shown from behind and lateral left with muscles (Mm. cricoarytenoidei post. and Mm. arytenoidei), the trachea, and the thyroid gland (Gl. thyroidea); D) a frontal section of a head shown from the front with the brain, the basilar artery as well as the ventricle and the vertebra exposed; E) a close up of the center with outer auditory canal (upper arrow), eardrum (middle arrow), and middle ear (lower arrow) exposed; F) a frontal section of a head viewed from the back, showing a cross section of the eyes, nasal cavities, paranasal sinuses and the oral cavity; G) a close up of the right eye with the lens and the ciliary body as well as an ethmoidal cell (upper arrow), nasal septum (middle arrow), and middle nasal concha (lower arrow) shown

In implementing the pilot project, the curricula of the dissection courses in the third and fourth semester were analyzed and specific module manuals detailing the content covered each day of the courses were compiled. Based on these manuals, the demand for plastinates was identified and compared to the inventory of existing specimens. A list of plastinates still required was compiled and ranked by priority. For the production of larger plastinated dissections additional larger equipment was purchased. Additionally, four 3D printed models were bought (see figure 1 (Fig. 1) for an example). Figure 2 (Fig. 2) shows examples of plastinates produced at the Centre of Anatomy.

For mediating the anatomical content various methods were used. Some methods, such as the demonstration of dissections materials (or plastinates) and models as well as teacher-centered direct instructions or lecturing, were identical to those in the regular dissection courses. Other strategies applied in the alternative courses included short student presentations on various topics, as a substitute for the students’ demonstration of their own dissection, as well as detailed discussions on the anatomical appearance, the topographical localization, and identification of structures during the dissection process. Apart from manual dissection skills, the alternative courses offer the students the same anatomical knowledge and skills as the regular dissection courses.

As the regular dissection courses, the alternative courses are supplemented with additional visual aids, such as bones, models, radiological images and illustrations. These courses are offered parallel to the regular ones with the same frequency and duration, requiring the involvement of an additional lecturer at the curricular level. Maintaining the same schedule for two courses means that students can switch to the alternative courses at any time if necessary.

#### 2.4. Examination and course evaluation

The alternative exams, to be sat at the end of the third and fourth semester, were designed to be identical, as much as possible, to the regular exams as described above. The oral exams were performed by lecturers that did not teach in the alternative courses. Results achieved in the two exams by the participating students were provided by the Office of Student Affairs and anonymized for evaluation in this study. Admission to the alternative courses and examinations is strictly limited. Students have to prove their pregnancy or that they are breastfeeding by a medical certificate. All other students attend the regular dissection courses.

To obtain feedback on the students’ experience in the various courses, Charité offers online feedback tools and requests the students to anonymously evaluate, for example, the dissection classes at the end of each curricular module.

For the alternative courses the Curricular Evaluation Unit designed a questionnaire which included performed rating statements with scales of agreement, but also allowed for additional comments. In the comments sections the students were asked to give positive and negative feedback on the general curricular conditions, the workload, the teaching and learning success and on the course in general (see attachment 1 : evaluation questionnaire, see attachment 2 : comments section). The data from the regular and the alternative courses were analyzed by the Curricular Evaluation Unit and made anonymously available after the end of each semester.

## 3. Results

### 3.1. Design and implementation 

The conceptualization of the alternative courses for the third and fourth semester took half a year of intense planning, structuring and cataloguing.

The compiled list of required plastinates was extensive, but mainly included speciments needed to supplement the existing collection and only a few others that were urgently needed. The list consisted of about 30 items, not including different stages of dissections (e. g. superficial and deep structures of extremities, heads, or situs, and variations/pathologies), series of axial sections of the extremities and situs, as well as organ clusters. The most urgently needed plastinates were produced first. There was a range of plastinates already available to provide adequate visual aids for many topics covered in the course. Therefore, the alternative course could be implemented as early as in summer semester 2017.

In adding necessary new plastinates that showed different stages of dissection, the opportunity arises to produce specimens illustrating anatomical variations, as well as pathologies found during dissection courses, which can be added to the inventory. These specimens will allow a better demonstration of the variability of anatomical structures and organs.

**Figure 3:**
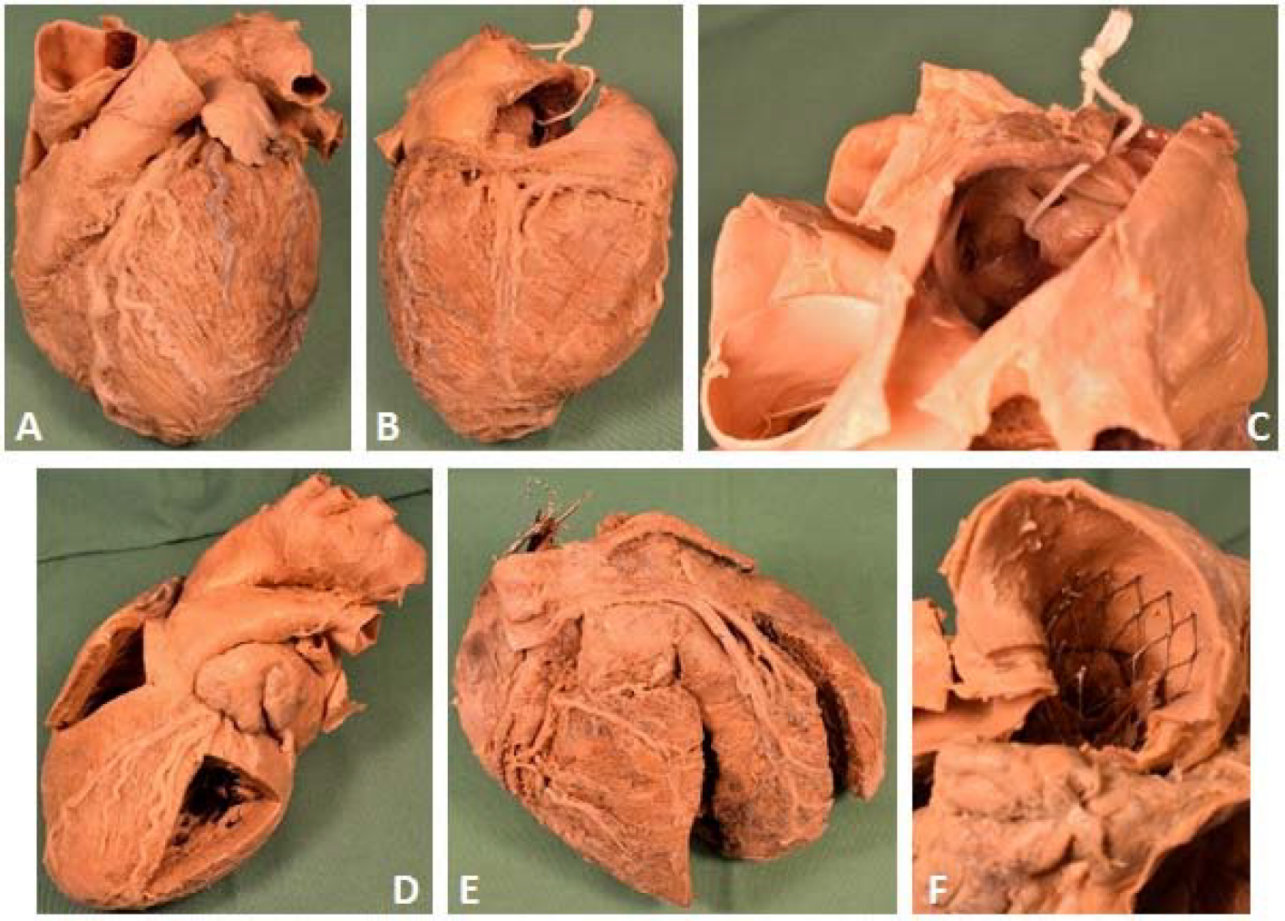
Examples of hearts plastinated at the Centre for Anatomy. A – C) a plastinated specimen of the heart viewed from the left (A), from the back (B) and a view of a patent foramen ovale with a ribbon pulled through it (C). D) A heart with fenestrated ventricles and the aortic arch with its major branches; E) a heart with bypasses and wires of a pacemaker; F) a stent with an artificial valve in the aorta

Furthermore, the plastinated specimens specifically produced for the alternative courses can be use in other courses and lectures. For example, a set of specimens produced with distinct anatomical and pathological variations of the heart anatomy, can also be used in a practical course of the regular curriculum addressing coronary heart disorders to illustrate healthy and pathological conditions (see figure 3 (Fig. 3)). Previously, pregnant and breastfeeding students were prevented from attending this practical course as FA-fixed hearts were used.

At least 5 students were enrolled in each course during the four semester pilot project, in which these alternative courses were offered (see table 2 (Tab. 2)). This means that at least every fortieth student enrolled in the third or fourth semester attended the alternative anatomical course.

#### 3.2. Evaluation

**Figure 4:**
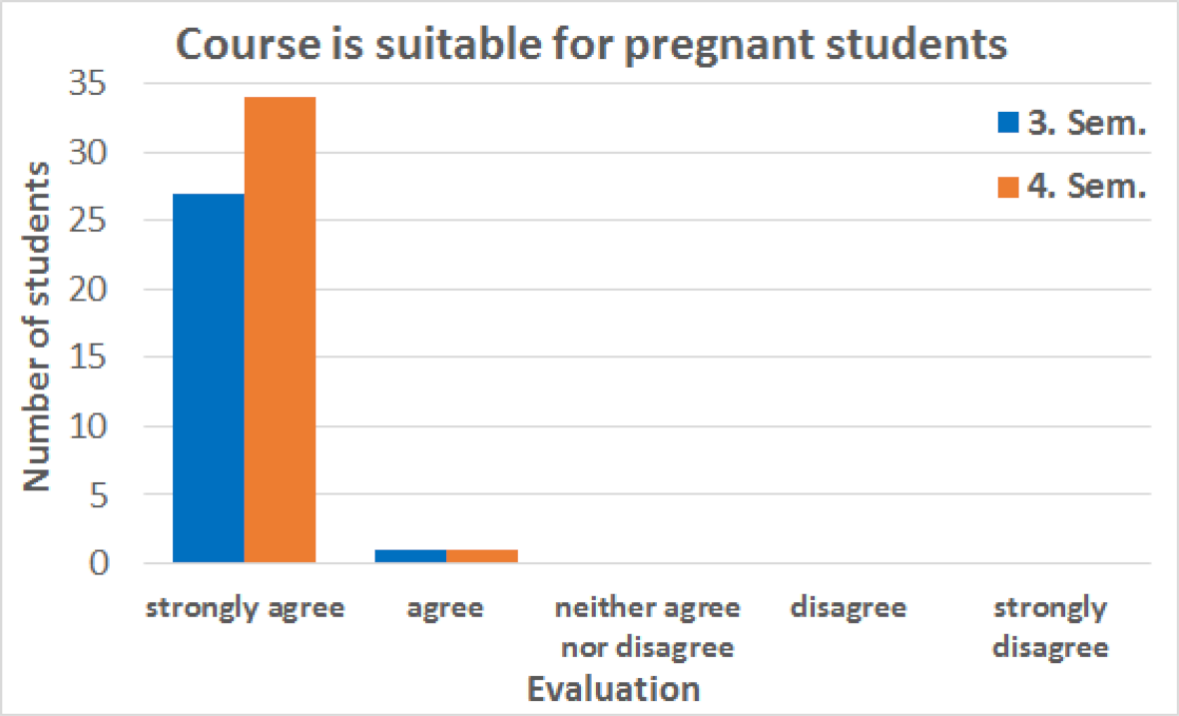
Feedback from students in third and fourth semesters (Sem.) regarding the statement “The design of the alternative course is compatible with a pregnancy (e.g. no long periods of standing).” In the third semester, of the 28 responses, a total of 27 participants strongly agreed with the statement and 1 participant agreed. In the forth semester, of the 35 responses to this statement 34 participants strongly agreed and 1 participant agreed. The median for both semesters is “strongly agree”. The students positively viewed the possibility to sit down during the course and put their feet up if necessary. They also liked not having to work in a bent or hunched over position for long periods as it is necessary when dissecting a cadaver. The data was collected from summer semester 2017 to winter semester 2018.

**Figure 5:**
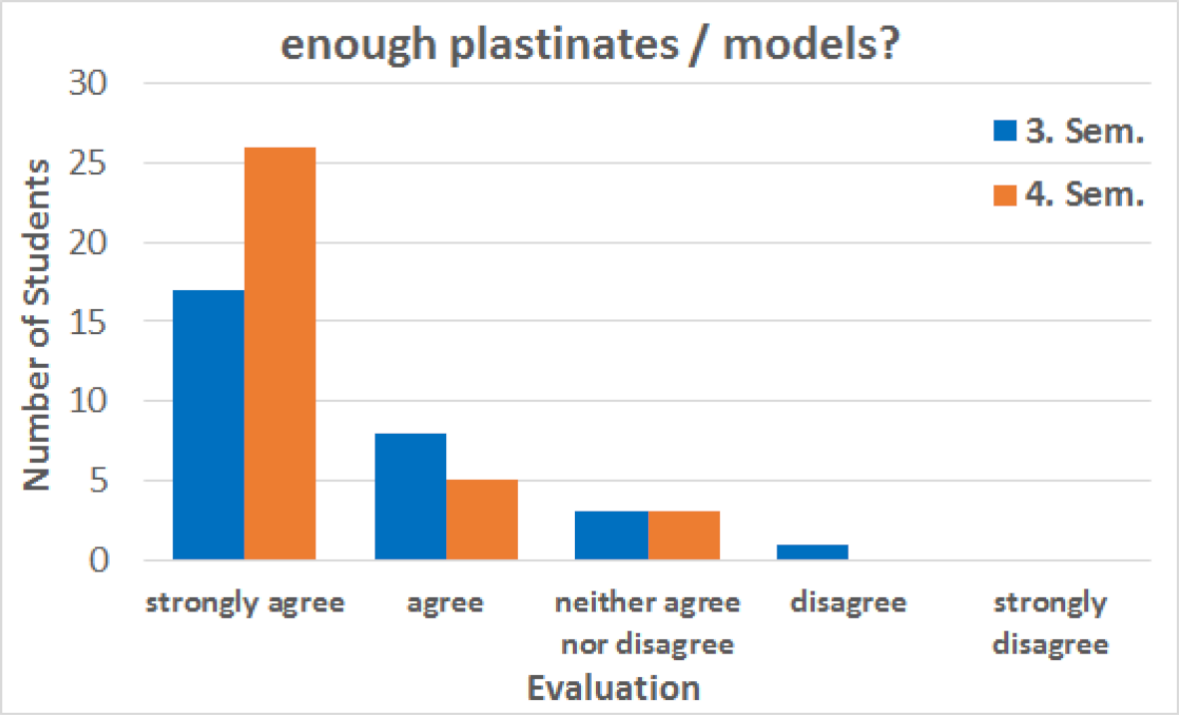
Feedback from students in the third and fourth semesters (Sem.) regarding the statement “There were enough plastinates/models available.” In the third semester, of the 29 responses to this statement 17 participants strongly agreed and 8 agreed. 3 participants stated that they neither agree nor disagree and 1 participant answered, “disagree”. In the fourth semester, of the 34 responses in total 26 participants strongly agreed, 5 agreed and 3 neither agreed nor disagreed. The median for both semesters is “strongly agree”. The data was collected from summer semester 2017 to winter semester 2018.

One expected result of the evaluation was that all participants agreed that the alternative course made it possible for them to move forward in their studies without delays. Another important question whether the alternative courses offered were compatible with a pregnancy was confirmed by the participation students (see figure 4 (Fig. 4)). In response to the statement that “enough plastinates/models were available” 58.6% of students in the third semester strongly agreed, 27.6% agreed and 10.3% neither agreed nor disagreed, while 3.4% did not agree. In the fourth semester, the majority of participants, 76.5%, strongly agreed while 14.7% agreed and 8.8% neither agreed nor disagreed (see figure 5 (Fig. 5)).

**Figure 6:**
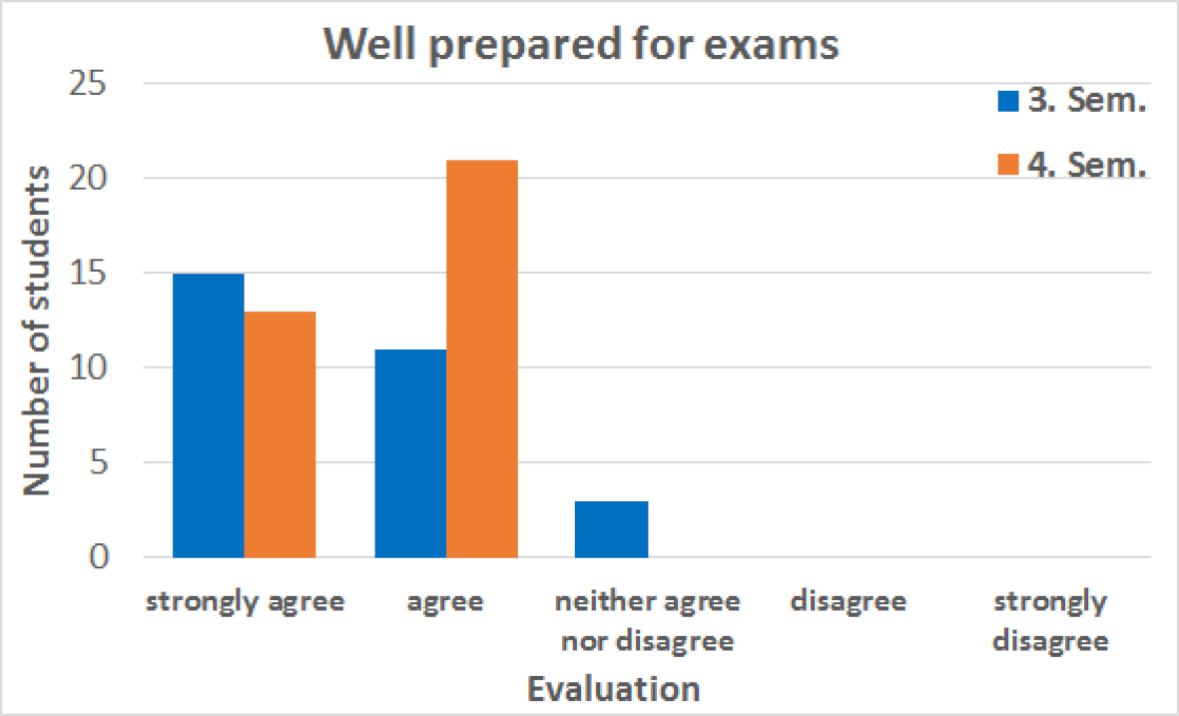
Feedback from students in the third and fourth semesters (Sem.) regarding the statement “After attending the alternative course I feel well prepared for the examination.” In the third semester, of the 29 responses, 15 participants stated that they strongly agree, 11 stated that they agree, and 3 participants neither agreed nor disagreed. In the fourth semester, of the 34 responses to this statement 13 participants strongly agreed and 21 agreed. The median for the third semester is “strongly agree” and for the fourth semester the median is “agree”. The data was collected from summer semester 2017 to winter semester 2018.

Responses to the statement that “the students felt well prepared for the examination after attending the alternative course” showed that in the third semester 51.7% of the students strongly agreed, 37.9% agreed and 10.3% neither agreed nor disagreed. In the fourth semester 38.2% of the participants agreed strongly and 61.8% agreed (see figure 6 (Fig. 6)). The distributed feedback questionnaires for the alternative courses were filled out by 53.6% of the participants in the third, and by 53.0% in the fourth semester.

#### 3.3. Examinations

**Figure 7:**
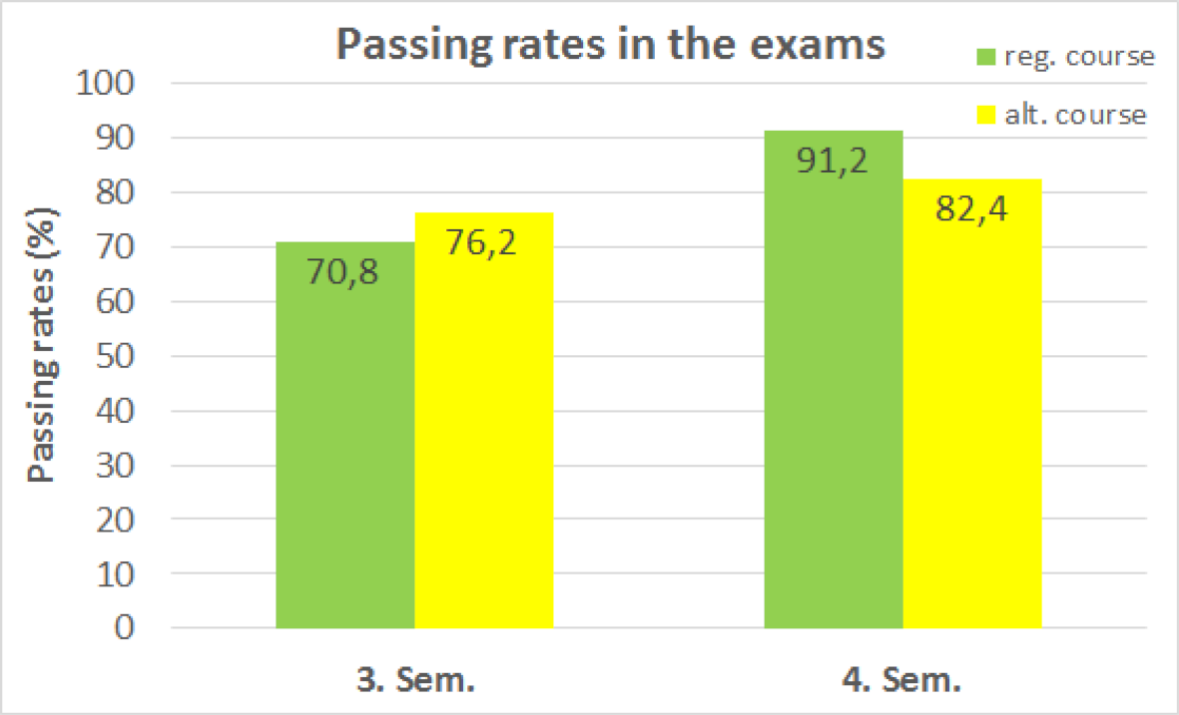
Passing rates among participants of the regular and the alternative courses. In the 3D-MC-examination at the end of the third semester (Sem.) a passing grade was achieved by 70.8% (579 of 820) of students attending the regular courses (reg. course) and by 76.2% (16 of 21) of the students attending the alternative courses (alt. course). At the end of the fourth semester, 91.2% (650 of 713) of the students attending the regular courses and 82.4% (14 of 17) of the students from the alternative courses passed the section on anatomy within the general oral examination. The data was collected from summer semester 2017 to winter semester 2018.

The implementation of the alternative exams raised no difficulties. In the 3D-MC examination the same questions were used as in the regular exam. Due to the broad range of plastinates collected by the time of the exams, suitable specimens could be assigned to most of the introductory questions even for the oral exams. The first results from the alternative exams indicate that the passing rates are approximately the same as those of the regular exams (see figure 7 (Fig. 7)). The average exam result of participants of the alternative course at the end of the third semester is 12.8 out of 20 points (64%; median: 13) and for students attending the regular course it was 13.0 points (65%; median: 13). In the examination at the end of the fourth semester students of the alternative course achieved an average of 71.9% (median: 78.3) and students of the regular course achieved 78.2% (median: 80.0). As students can choose when to sit the exam and because they can also attend a semester multiple times, the number of students taking the exams is lower than that of course participants.

## 4. Discussion

Prosection or the more in-depth dissection course are well-established elements of medical education [1], [2], [3]. Efforts are being made to teach students about anatomical structures without the use of body donors [42]. Over the last 240 years wax models offered the possibility to illustrate anatomical details to whole student cohorts and even generations of medical students. The detailed Florentine wax models, exhibited in the Josephinum in Vienna, are proof of this fact [42], [43]. Today, probably every institute of anatomy uses various models.

However, regular dissection courses serve not only to teach the anatomical structures of the body. Students cite various advantages, such as a form of preparation for their clinical work, being able to apply practical skills, work in a team, to combine theory and praxis [5], getting support in professional development and in handling the topics of dying and death [44]. A majority of students claims to feel better prepared for exams and to have a more precise understanding of study topics [45].

Studies show that students see dissection courses as an important resource for learning about human anatomy, topography and variations, rating it above lectures and textbooks. Multimedia as a learning tool is less popular [11], [46]. When it comes to knowledge transfer, 3D software applications are viewed inferior to real anatomical models [29], [34]. Dissection courses have been proven to be effective and popular [5], [11], [44]. Students describe it in both positive (interesting, fascinating, useful, brilliant, …) as well as negative terms (unpleasant smell, intimidating, repulsive, unreal, …) [5], [8].

### 4.1. Alternative teaching strategies

By the end of the 1980s digital media were tested as supplementary teaching material. The Ohio State University, for example, compared the learning success of students who saw video recordings of anatomical demonstrations, to that of students attending prosection courses on the same topic. The average results of both groups were almost identical [6]. According to a recent study, however, the majority of students seldom use videos that were made available to them to prepare for courses [27]. To improve radiological understanding, virtual dissection tables have been introduced [25]. In Tübingen dissection courses are successfully complemented by “obligatory curricular live stream”-lectures to improve the students’ clinically-relevant anatomical understanding [47].

#### 4.2. Courses, examination, and evaluation

The use of plastinates – here described for the implementation of the revised Maternity Protection Act – is another possible approach for teaching anatomy. Evaluation results show that courses can be successfully implemented provided that a sufficiently large set of plastinates are available. These FA-free courses are particularly suitable for pregnant students, not only because they can work sitting down. The subjective feeling that students consider themselves well prepared for the exams has been confirmed by the first examination results, albeit the low number of participants. In the exam after the third semester, the same questions as those in the regular exams can be used and in the examination after the fourth semester the introductory question can be taken from the catalogue for the regular exams that is available to all students. This allows for a high level of correspondence in the examination conditions for all students regardless whether they are taking the alternative or the regular exam. All questions are checked and approved by a committee of expert representatives and/or the board of examiners. In both the 3D-MC and the oral exams, five groups of students are tested simultaneously in parallel exam parcours. Therefore, the differences include the use of corresponding, but not fully identical anatomical specimens, as well as the involvement of different examiners. The participants of the alternative courses achieved similar results as their fellow students, even though they were expected to have disadvantages while studying due to a lack of sleep and reduced ability for concentration during their pregnancy or while breastfeeding. How long lasting the learning effect in the regular and the alternative courses is, would have to be explored in elaborate follow up studies in the coming years.

At Warwick Medical School a study looked at how students perceive the use of plastinates for anatomical education [37]. A majority of 94% of study participants evaluated plastinates as a valuable resource for learning. The participants highlighted various positive aspects, such as the excellent anatomical detail and the depiction of the topographic relationship between structures. The study also showed that learning with plastinates alone limits the aspect of tactile perception and the emotional experience. Additionally, it seems that the understanding of topographic relationships is limited as the organ clusters and the dissected situs lack flexibility. One of the major advantages of plastinates, on the other hand, is that anatomical structures which, due to time restrictions, are not or only partly dissected in regular courses can be presented and preserved.

Students had the opportunity to give feedback in a uniform, official and transparent evaluation questionnaire. No other methods of evaluation were used. Generally, students at Charité are rather unmotivated to give feedback (2.2% or 2.8% in the regular dissection courses). The response rate in the alternative courses is markedly better, probably as a result of reminders to the students by the lecturer. Verbal feedback given directly to the lecturer has been only positive.

The alternative courses offer a major advantage to the students by facilitating a seamless continuation of their studies, instead of completing the dissection courses and related exams at a substantially later date. The aim of this pilot project was to provide comparable conditions, as far as possible, for learning human anatomy to pregnant and breastfeeding students as experienced by their fellow students. Considering the feasibility of the implementation the results of the exams and evaluations, we believe that this aim has been achieved.

#### 4.3. Transferability

The successful implementation of the Maternity Protection Act described here, may work less efficiently at smaller medical universities. With smaller student cohorts, fewer pregnant and breastfeeding students would be wishing to attend the courses. The financial burden would include the position of a lecturer, the costs for storaging the plastinates and if needed, the commercial acquisition of plastinates, estimated at about 500,000 € (based on quotes from a manufacturer). The interpretation of the law, as to when the introduction of a course for pregnant and breastfeeding students is considered to be unfeasible, is the universities’ responsibility and the disproportionate effort involved needs to be verifiably demonstrated. Individual circumstances will be the decisive factors. Transferring this concept of an alternative course to educational settings in other fields of medicine (e.g. practical training in biochemistry, pathology or bedside teaching) is highly questionable as the circumstances and demands are too distinct. Additionally, the affected courses mostly involve much fewer teaching units. As other colleagues, we at the Centre for Anatomy at Charité agree that teaching human anatomy on human and flexible cadavers leads to the most sustainable learning results [6], [31]. About 90% of medical students consider a deep and thorough understanding of human anatomy as very important [37]. For many future medical professionals, however, it is not essential to dissect a body themselves for their perspective medical work, nevertheless, this experience will increase their abilities to understand the what, the why and the how of medical interventions with high relevance for patient safety as well as for their own satisfaction as physicians [14]. Computer programs and virtual technologies can and should support anatomical education. However, these cannot substitute the experience obtained in a dissection course. Therefore, dissection courses should be attended if at all possible. A thorough understanding of anatomical relations in the body is essential for future physicians and has to be taught very well.

## 5. Conclusion

The newly developed alternative macroscopic anatomy courses and exams for pregnant and breastfeeding students constitute adequate alternatives closely matching the regular dissection courses in the medical curriculum. This method is recommended to all institutes that have the appropriate resources and opportunities. The number of participants shows that there is enough demand to warrant the resources and efforts required to plan and implement such courses. The project at Charité was extended after the pilot phase and is running now as an integral part of the curriculum. The introduction of similar alternatives for anatomical courses in later semesters is in discussion.

In view of the well established advantages of regular dissection courses and the limitations of their alternatives, we recommend dissection courses as the central elements in the anatomical education of medical students. At the same time, we recommend the here described alternative macroscopic anatomy courses which uses plastinates, as a valid and useful substitute program for pregnant and breastfeeding students.

## Acknowledgements

We want to thank our colleagues at the Office of Study Affairs for their support and for providing the anonymized exam results after the third and fourth semester and the team at the Curricular Evaluation Unit for designing the evaluation questionnaires and for providing the anonymized evaluation results. We also thank the Vice Deans for Teaching and Learning at Charité, Prof. A. Kuhlmey and Prof. J. Spranger, for their continued support in this project.

Finally, a big thank you to all the dedicated people who decided to become body donors and support medical education.

## Competing interests

The authors declare that they have no competing interests. 

## Supplementary Material

evaluation questionnaire

Open-answers – questionnaires

## Figures and Tables

**Table 1:**
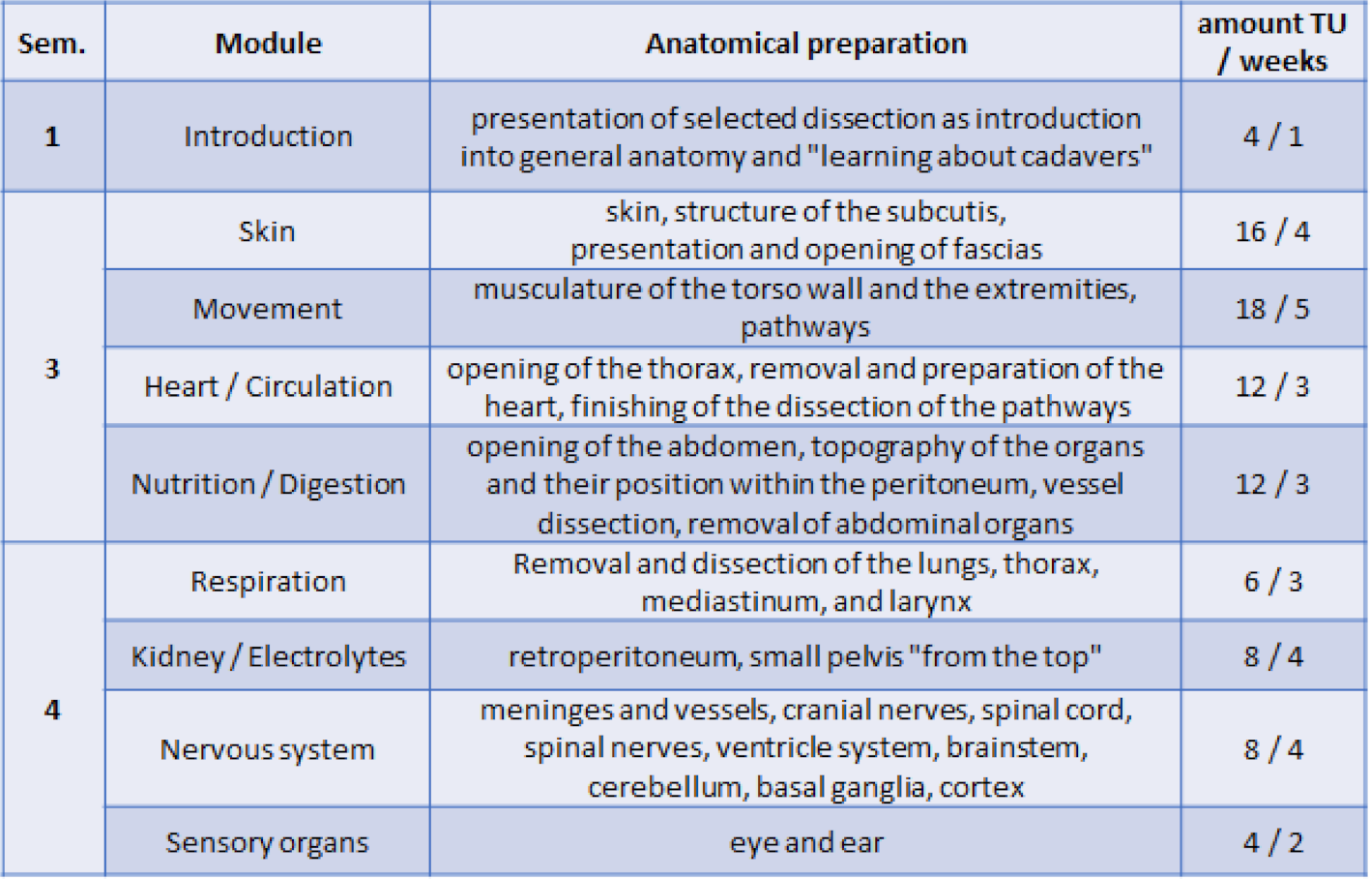
Current schedule for anatomic dissection in semester 1 to 4 of the model degree program for medicine at Charité – Universitätsmedizin Berlin. This table shows the distribution of the major elements of anatomical dissection in the semesters (Sem.) and the curricular modules. It also shows the number of teaching units (TU) for dissection in each module as well as the length of the modules in weeks.

**Table 2:**
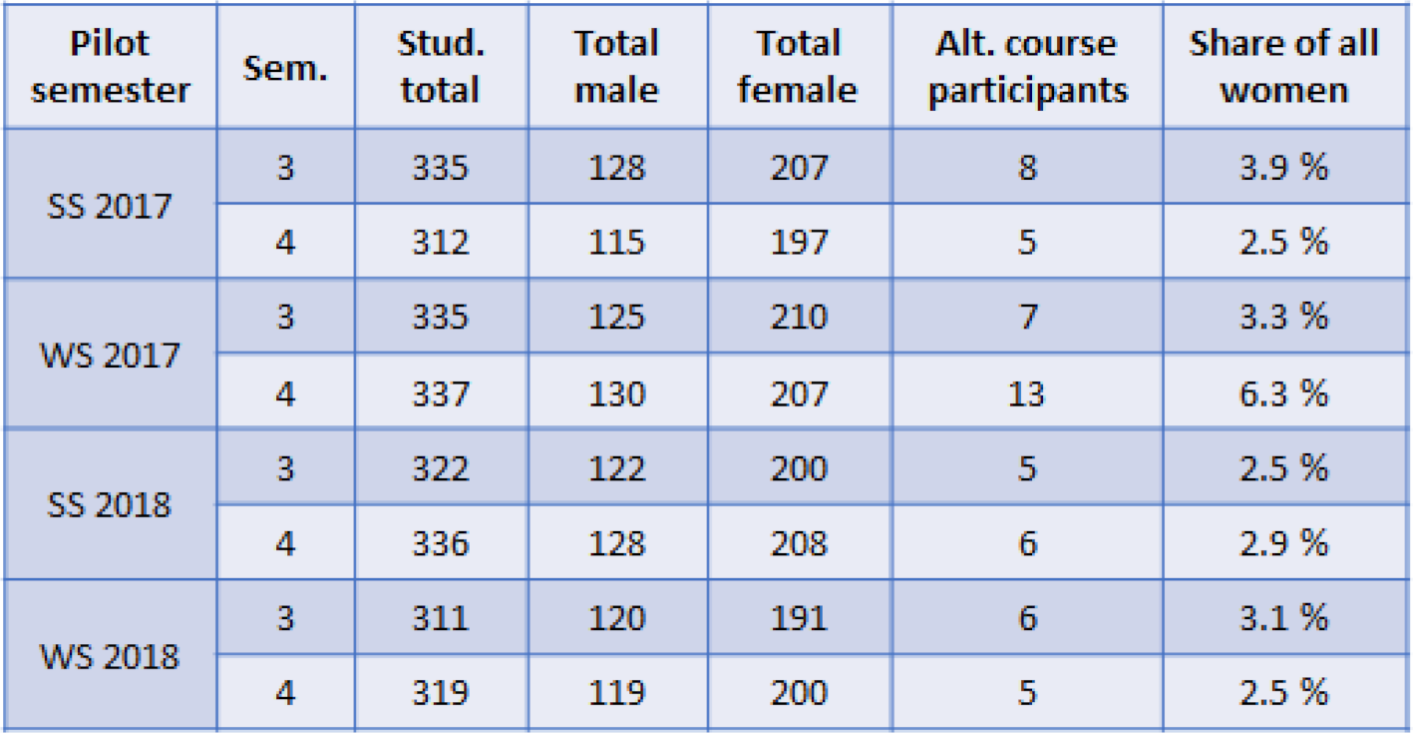
Number of course participants in the third and fourth semester (Sem.) of the model degree program of medicine at Charité – Universitätsmedizin Berlin. In all four pilot semesters (summer semester 2017 – winter semester 2018) the share of female students was about 60% of the students cohort. In the third semester, 1 in 40 to 1 in 26 female students attended the alternative course. In the fourth semester, 1 in 40 to 1 in 16 female students attended the alternative course. Before the introduction of the alternative courses, students had to wait until after their pregnancy or the breastfeeding period (up to four semesters and more) to attend the dissection courses. Following the introduction of the new alternative courses, in the first two pilot semesters these students attended the courses alongside the pregnant and breastfeeding students attending there respective semester. Over the course of the winter semester 2017 another five students in the fourth semester became pregnant and transferred from the regular course to the alternative course.
