# Supplementary material for: Challenges and implementation of the German maternity protection act for female medical students in macroscopic anatomical education
Source: GMS J Med Educ. 2020 Mar 16;37(2):Doc17. doi: 10.3205/zma001310 (PMC7171363; doi:10.3205/zma001310)
Supplement: evaluation questionnaire [file JME-37-17-s-001.pdf]

EvaSys	Macroscopic anatomy for pregnant and breastfeeding students.Third Semester	Electric Paper

Charité - Universitätsmedizin Berlin • Dean for Teaching and Learning • Devision for Quality Management

Dissection course for pregnant and breastfeeding students – 3. Semester

Qualitätssicherungsbereich

Mark like this: ☐ ☒ ☐ ☐ ☐ Please use a ballpoint pen or a light felt tip pen. This questionnaire will be processed by machine.

correction: ☐ ■ ☐ ☒ ☐ To ensure optimal data processing please ensure to take notice of the notes on the left.

Curricular framework conditions (course content, content structure, learning spiral)

Dear students, your answers are collected and processed anonymously. In small groups, however, your answers could indicate your identity. Therefore, this evaluation is voluntary.

I am evaluating the alternative dissection course in module: ☐ M09 ☐ M10 ☐ M11 ☐ M12

	strongly agree	agree	neither agree nor disagree	disagree	strongly disagree
The content of this course was well structured.	☐	☐	☐	☐	☐
The dissection course fitted well into my schedule ...					
... in regard to time.	☐	☐	☐	☐	☐
... in regard to its content.	☐	☐	☐	☐	☐
There were enough plastinates/models available.	☐	☐	☐	☐	☐
This alternative dissection course compared to having to take time off has saved me time.	☐	☐	☐	☐	☐
The design of the alternative course is compatible with a pregnancy (e.g. no long periods of standing).	☐	☐	☐	☐	☐

	far too high	too high	appropriate	too low	far too low
The level of difficulty of the course content was ...	☐	☐	☐	☐	☐

curricular framework conditions: I liked the following:

Attachment 1 to: Kulisch C, Langheinrich J, Heuckendorf E, Vida I, Brunk I. Challenges, and implementation of the German maternity protection act for female medical students in macroscopic anatomical education. GMS J Med Educ. 2020;37(2):Doc17. DOI: 10.3205/zma001310, URN: urn:nbn:de:0183-zma0013107
Online available from: https://www.egms.de/en/journals/zma/2020-37/zma001310.shtml

Curricular framework conditions (course content, content structure, learning spiral) [continuation]

Curricular framework conditions: I would improve the following:

students (workload, participation, interaction)

	a lot more	more	the same amount	less	a lot less
compared to my fellow students attending the regular dissection course, I believe to have learnt ...	☐	☐	☐	☐	☐

	strongly agree	agree	neither agree nor disagree	disagree	strongly disagree
I had the necessary previous knowledge for this course:	☐	☐	☐	☐	☐

The following question is important for us, even though it might compromise your anonymity. We ask you to voluntarily answer it:
I attended the alternative course because of the following reasons (multiple answers possible):

☐ pregnancy ☐ breastfeeding ☐ health related reasons

students: I liked the following:

Attachment 1 to: Kulisch C, Langheinrich J, Heuckendorf E, Vida I, Brunk I. Challenges, and implementation of the German maternity protection act for female medical students in macroscopic anatomical education. GMS J Med Educ. 2020;37(2):Doc17. DOI: 10.3205/zma001310, URN: urn:nbn:de:0183-zma0013107
Online available from: https://www.egms.de/en/journals/zma/2020-37/zma001310.shtml

students (workload, participation, interaction) [continuation]

students: I would improve the following:

teaching and studying success (promotion of interest, increased skills)

	strongly agree	agree	neither agree nor disagree	disagree	strongly disagree
The amount of content tested in the progress quizzes was appropriate.	☐	☐	☐	☐	☐
After attending the alternative course I felt well prepared for the examination.	☐	☐	☐	☐	☐

teaching and studying success: I liked the following:

teaching and studying success: I would improve the following:

Attachment 1 to: Kulisch C, Langheinrich J, Heuckendorf E, Vida I, Brunk I. Challenges, and implementation of the German maternity protection act for female medical students in macroscopic anatomical education. GMS J Med Educ. 2020;37(2):Doc17. DOI: 10.3205/zma001310, URN: urn:nbn:de:0183-zma0013107
Online available from: https://www.egms.de/en/journals/zma/2020-37/zma001310.shtml

overall evaluation

	very happy	happy	neither happy nor unhappy	unhappy	very unhappy
Overall I am … with the course.	☐	☐	☐	☐	☐

I liked the following aspects of this course:

I would improve the following aspects of this course:

Attachment 1 to: Kulisch C, Langheinrich J, Heuckendorf E, Vida I, Brunk I. Challenges, and implementation of the German maternity protection act for female medical students in macroscopic anatomical education. GMS J Med Educ. 2020;37(2):Doc17. DOI: 10.3205/zma001310, URN: urn:nbn:de:0183-zma0013107
Online available from: https://www.egms.de/en/journals/zma/2020-37/zma001310.shtml
