# Supplementary material for: Challenges and implementation of the German maternity protection act for female medical students in macroscopic anatomical education
Source: GMS J Med Educ. 2020 Mar 16;37(2):Doc17. doi: 10.3205/zma001310 (PMC7171363; doi:10.3205/zma001310)
Supplement: Open-answers – questionnaires [file JME-37-17-s-002.pdf]

Attachment 2: Open-answers – questionnaires

The following is a list of the comments in the evaluation questionnaires.

3. Semester

Curricular framework conditions: I liked the following

- being able to bring my child with me when I'm in a pinch
- great prosection specimens and models
- great integration into the lesson plan
- linked well to other courses
- topics presented in lectures are integrated and revised on models
- Thanks to the models, cross sections, plastinates and what the lecturer described from the dissection lab I felt well informed on the content and successfully integrated in the learning spiral.
- the order of topics was well structured
- the short presentations are a very good idea
- the illustrative material was great, all questions were answered, nice atmosphere
- well structured, all clarification questions were answered
- the lecturer is very eager to bring the topics/content across
- the use of visual aids (plastinates, x-ray imaging, etc.)
- the short student presentations

Curricular framework conditions: I would improve the following

- more plastinates, dissections
- plastinates of the abdomen/thorax
- For me personally, the rotations of the dissection course collided with my other courses, which made it quite difficult for me.
- There is so much to learn in anatomy in the third "Fachsemester" that I wish there would have been more revision sessions and sessions with exam-oriented quizzing.

Attachment 2: *Kulisch C, Langheinrich J, Heuckendorf E, Vida I, Brunk I. Challenges, and implementation of the German maternity protection act for female medical students in macroscopic anatomical education. GMS J Med Educ. 2020;37(2):Doc17. DOI: 10.3205/zma001310, URN: urn:nbn:de:0183-zma0013107*
Online available from: https://www.egms.de/en/journals/zma/2020-37/zma001310.shtml

- Personally, I think that the councils with the lymphatic nodes are not that important or they are not covered enough during the courses.
- I actually had no fun at all.

Students: I liked the following

- to be able to compare different plastinates, e. g. of the heart
- relaxed and friendly atmosphere
- a lot of opportunity to talk about experiences
- excellent opportunity to talk to the other students and the lecturer
- everyone was very friendly and polite
- as there are many topics to learn: a lot of revisions → amazing increase in knowledge
- many opportunities to clarify questions
- topics were discussed extensively
- I think the student presentations are very useful as students automatically dive deeper into the topic.
- great atmosphere for studying
- nice atmosphere, mutual support
- everyone was very friendly with one another, intimate environment, individual needs were taken into account
- The lecturer made sure everyone learned the same and achieved a high level of understanding.
- great group size
- lecturer kept to the timeframe, always well prepared, great group size
- The small group made active participation possible and encouraged it. I liked how the topics for the presentations were divided up – very sensible.

Students: I would improve the following

- during the course less talk about babies, breastfeeding, birth etc.
- group tasks and presentations would be great
- homework from one day to the next is difficult to realise
- a bit less time spent on muscles, more time for other organs
- plan for more time on the heart, less revision of the muscles

Teaching and studying success: I liked the following

- regular lectures/presentations
- the student presentations were an opportunity to prepare a topic in-depth and impart your knowledge to the other students → great opportunity to check your study progress and your level of understanding
- revision of the topics of the previous course day → to test your knowledge
- Presentation on a topic that you might not like and/or care for, or a topic you are unfamiliar with → the presentation is an opportunity to review your level of understanding
- revision → deepen your knowledge
- I felt like I had already learned a lot after each session, as we discussed the anatomical structures in such great detail
- there was enough time to discuss all topics as long as was necessary for everyone to understand them
- Because of the short student presentations students were well prepared for topics
- revision quizzes at the beginning of the session
- the content of the previous session was often revised in the next session

Teaching and studying success: I would improve the following

- It would have been great if the lecturer had been a bit stricter so that I and others could have prepared and followed-up better.
- Checks or rather quizzes about what was learnt in the form of MC questions to be able to review your own progress after each module and discuss potential difficulties.
- potentially more quizzes for self-monitoring
- short presentations
- Exam-oriented quizzing rounds would help a lot.
- The short presentations were way too long, precise instructions on what should be covered in the presentations would be better.

Overall evaluation: I liked the following aspects of this course

- short student presentations
- revision of the previous session
- module manuals as guidelines
- nice and polite learning environment
- engagement and empathy of the lecturer
- detailed study of anatomy
- the nice interaction with the lecturer and fellow students
- the opportunity to attend a dissection course and take the 3D-MC while pregnant or breastfeeding – thank you!
- more time to discuss theoretical anatomy and talk about the function of certain structures
- detailed answers to all questions
- questions were answered in detail by the lecturer and fellow students
- being able to talk about problems with certain anatomical structures
- practical application
- cross sections showing where something could be (only seen in books before)
- very polite and motivated lecturer
- great studying success
- not anxious before the exam
- it feels great not to be excluded due to pregnancy and/or breastfeeding
- because of the small group size everyone could be catered for
- everyone had a chance to present and/or explain something
- very useful
- Obviously a lot of effort was put into the planning and implementation of the course
- motivated and eager lecturer; in general, to have the offer or rather opportunity to not loose time during the pregnancy and the breastfeeding period because of the alternative course
- motivated and patient lecturer; illustrative material (photos, x-ray imaging, dissections); quizzes for self-monitoring
- the lecturer was very eager and left no question unanswered

Overall evaluation: I would improve the following aspects of this course

- time of the courses (morning sickness, getting the kids ready in morning takes time)
- more plastinates
- move the course back a bit to 10:15 AM so it does not collide with parental duties
- There were enough plastinates or models. In case there are other methods available (e.g. transverse sections), it would be nice to see those too

4. Semester

Curricular framework conditions: I liked the following

- well integrated into the course plan and linked well to other lectures, topics presented in lectures are revisited and revised on models etc. +
- Fits well with the lesson plan as the course was at the beginning of the week. Gave you a great overview of the topic of each week's seminars and hands-on training.
- This was illustrated on models, cross sections, plastinates and, additionally, by the lecturer.
- the structured approach
- strictly oriented to the learning objectives
- You never feel disadvantaged for attending "only" the alternative course. On the contrary, I even thought there were more opportunities for knowledge exchange.
- great revision of the entire module (16)
- great models
- Everything we discussed was relevant to the examination and fitted well into the learning objectives.
- The topics related very well to the learning objectives.
- the revision of the topics, deepening of knowledge. I also liked the plastinates. They were very instructive.
- The many plastinates and other visual aids (power points) made it easy to follow. This meant you learnt a lot.
- the visual material was very good, too
- learning spiral is visible, as questions of earlier modules were clarified and topics revisited

Curricular framework conditions: I would improve the following

- The exams require so much knowledge that I think it would be useful to have an online quiz for self-monitoring (e.g. mock exam questions) after each module of the alternative as well as the regular dissection course.
- more plastinates

Students: I liked the following

- Fewer students, a lot of opportunities for exchange
- Fewer students, which means you learn a lot more because more is demanded of you
- great opportunity to talk to your fellow students and the lecturer
- everyone was very nice and polite
- the sessions were varied (power point presentations, lectures, each session had fitting plastinates, panels)
- communication between lecturer & students
- I liked that we had no presentations. I don't mind the effort of making one, but I find it more effective working on the topics in the group **note: due to time restrictions there was no short presentations in two modules**
- I liked that the lecturer voiced his expectations; this meant that everyone was always well prepared and did their follow-up. At the beginning of each session the topics of the previous session were revised. There were enough opportunities to ask questions and close knowledge gaps.
- very capable lecturer
- opportunities for questions, great working and studying atmosphere
- enough time for clarifying questions

Students: I would improve the following

- I would like group work and group presentations
- some presentation topics could not be covered in 5 minutes
- some of the presentation topics were too broad for a 5-minute talk
- regular short student presentations can be useful to ensure everyone participates
- in part the participation frequency was very varied
- Too little time for follow-up and exchange on the topic areas especially when you have a toddler or baby at home.

Teaching and studying success: I liked the following

- The student presentations were an opportunity to prepare a topic in-depth and impart your knowledge to the other students, which was a great opportunity to check your study progress and your level of understanding. The revision of the last session at the beginning of each session → self-monitoring of knowledge

- Student presentation gave an opportunity to prepare for one topic more extensively and the actual presentation was an opportunity to test whether you can actually get your knowledge across (good for the exam).
- link to the other sessions, partly through revisions that deepen knowledge
- dividing the study content up into presentations
- the practical training after module 16 was a great opportunity to test your knowledge
- the learning spiral was integrated
- existing knowledge was combined well with new knowledge
- individual mentoring of the students
- seeing and handling the different dissections/models made it easy to understand everything
- The lecturer was great. Especially his technical knowledge and the interesting information on pathology with surgical techniques.
- It is nice how everyone treats each other respectfully:
- Thank you for this great learning opportunity.
- Questions were answered in-depth and with a lot of expertise.
- The review of the last session at the beginning was amazing.
- there was enough time to discuss the wider context of things, elaborate on the comparison of sensory organs

Teaching and studying success: I would improve the following

- At the end of each module there could be a short quiz with MC-questions to test how much you learned. Then potential difficulties could be revised in group.
- You could work on the presentations during a session with the models that are available and the anatomical books. E. g. 30 minutes preparation time, then the presentation etc.
- a bit more feedback in regard to the level of knowledge would be great → to test your study Progress

- more obvious tests for checking our progress, more dissections, a bit more illustrative material
- quizzes after each module

Overall evaluation: I liked the following aspects of this course

- enough time for the theoretical anatomy and for discussing the function of specific structures, questions could be answered in detail
- short student presentations
- revision of the topics of the previous session
- module manuals as guideline(s)
- nice and polite studying environment
- the dedication and empathy of the lecturer
- the opportunity to ask questions and get them answered
- detailed explanations
- handling of cross sections, which show what could be were, and on what level, etc.
- thanks to the manual for the alternative dissection courses you could prepare well for the up-coming session
- selection of dissections/models was very good in my opinion
- I can compare the "normal" course to the alternative dissection course and I feel like I learned the contents a lot more in-depth when attending the alternative course. Thanks to the intensive work with books and the available plastinates you could work well on what you are expected to learn. I am very happy to have the opportunity to attend the alternative course, so that I don't fall behind in my studies! Without this course I would not have been able to continue my studies next semester.
- great explanation, preparation
- great models, cross sections, plastinates
- Thank you for the great course!
- Very instructive course and great learning atmosphere!
- motivating lecturer
- very qualified teaching, great models

Overall evaluation: I would improve the following aspects of this course

- move the course back a bit to 10:15 AM so it does not collide with family duties
- time of the courses (morning sickness, getting the kids ready in morning takes a bit more time after all)
- 8:15 AM is very difficult as our nursery only opens at 8 AM. Maybe it’s possible to move the course back.
- regular testing of the learning progress as a comparison standard to the normal dissection courses
- more frequent checks on the learning progress
